# Effect and mechanism of poly (ADP-ribose) polymerase-1 in aldosterone-induced apoptosis

**DOI:** 10.3892/mmr.2015.3596

**Published:** 2015-04-07

**Authors:** WEIWEI QIAO, WEILI ZHANG, SHUHONG SHAO, YUSHENG GAI, MINGXIANG ZHANG

**Affiliations:** 1 Department of Diagnostics, Binzhou Medical University, Yantai, Shandong 264003, P.R. China; 2 Department of Cardiology, Yantaishan Hospital, Yantai, Shandong 264000, P.R. China; 3 Department of Medical Psychology, Binzhou Medical University, Yantai, Shandong 264003, P.R. China; 4 Key Laboratory of Cardiovascular Remodeling and Function Research, Chinese Ministry of Education and Chinese Ministry of Public Health, Shandong University, Qilu Hospital, Jinan, Shandong 250012, P.R. China

**Keywords:** atherosclerosis, aldosterone, aldosterone receptor antagonists, polyadenylation diphosphate ribose transferase 1, apoptosis, endothelial cells

## Abstract

The present study aimed to investigate the effects of aldosterone on vascular endothelial cells and the viability of poly (ADP-ribose) polymerase 1 (PARP1) in cells, and to examine the molecular mechanisms underlying the effects of aldosterone on vascular endothelial cell injury. Cultured endothelial cells were treated either with different concentrations of aldosterone for the same duration or with the same concentrations of aldosterone for different durations, and the levels of apoptosis and activity of PARP1 in the cells were detected, respectively. Aldosterone receptor antagonists or PARP1 inhibitors were added to cells during treatment with aldosterone and the levels of apoptosis and activity of PARP1 were detected. As the concentration of aldosterone increased or the treatment time increased, the number of apoptotic cells and the activity of PARP1 increased. The aldosterone receptor antagonists and PARP1 inhibitors inhibited the increase of apoptosis and PARP1 activity caused by aldosterone treatment. Aldosterone activated the activity of PARP1 via the aldosterone receptor, inhibiting cell proliferation and inducing apoptosis. Treatment with PARP1 may be used as a target for vascular diseases caused by aldosterone at high concentrations.

## Introduction

The role of aldosterone in cardiovascular disease has received increasing attention (1–8). It has been reported that elevated concentrations of plasma aldosterone cause endothelial dysfunction and trigger a series of vascular lesions (9), however, the underlying molecular mechanism remains to be elucidated. Poly (ADP-ribose) polymerase (PARP)1 is a nuclear localization protein with a molecular weight of 116kDa, and has low levels of activity in normal cell growth (10–12). The predominant function of PARP1 is in single strand DNA damage repair, to avoid changing the genetic information of cells (10–12). When DNA damage occurs, PARP1 is activated to repair the DNA and, if the damage increases further, PARP1 is fully activated consuming high levels of energy, eventually leading to cell death (13,14).

A previous investigation found that PARP-1 is important in a variety of cardiovascular disease processes (15) and that the catalytic activity of PARP-1 increases significantly in the development of these diseases. Previous studies, using animal models of cardiovascular disease, demonstrated that PARP inhibitors have a positive and effective role in alleviating the symptoms of cardiovascular disease (14). However, the underlying molecular mechanism remains to be elucidated. It was hypothesized that changes in the activity of PARP1 involved in the apoptosis of endothelial cells were caused by aldosterone. In the present study, primary cultured human umbilical vein endothelial cells (HUVECs) were selected as models of endothelial cells. The cultured cells were treated with aldosterone for identical durations or at the same concentration for different durations and the levels of proliferation and apoptosis were assessed. The PARP1 activity in the cells was simultaneously detected.

Eplerenone is a novel, selective aldosterone receptor inhibitor with advantages, including low levels of interaction with androgen and progesterone receptors, longer half-life, low incidence of side effects and good tolerance, compared with other aldosterone receptor inhibitors (16,17). Once daily oral administration effectively controls high blood pressure and reduces damage to the heart, brain, kidney and other target organs. Since its introduction, eplerenone has demonstrated the ability to control hypertension, prevent target organ damage associated with cardiovascular disease and improve the prognosis of patients with hypertension (18–20).

To ascertain whether the damage caused by aldosterone on endothelial cells derives from the direct effects of aldosterone on aldosterone receptors, eplerenon, an aldosterone receptor antagonist with fewer side effects and improved specificity, was selected to treat cells in combination with aldosterone. The present study aimed to determine whether eplerenone was involved in offsetting the endothelial cell apoptosis and increased activity of PARP1 caused by the extracellular signaling molecule, aldosterone and, ultimately, to investigate how aldosterone affects intracellular apoptosis and PARP1 activity.

## Materials and methods

### Cell preparation

HUVECs were collected from the neonatal umbilical cord by perfusion digestion (Yantaishan Hospital, Yantai, China) according to a previously reported method (21). The cells were cultured in RPMI-1640 complete medium (Shanghai Sangon, Biological Engineering Co., Ltd., Shanghai, China) and then were incubated in a culture flask (1×10^6^/ml), followed by culture in 5% CO_2_ and at 37°C. The medium was refreshed every 24 h. Once the growth density of primarily cultured cells reached more than 80%, the culture fluid was discarded. Following washing with phosphate-buffered saline (PBS; Sigma-Aldrich, St. Louis, MO, USA) for 2–3 times, 0.25% trypsin (Fuzhou Maixin Biotechnology Development Co., Ltd., Fuzhou, China) was added for digestion at 37°C for 1 min. The mixture was centrifuged at 200 x g for 5 min. The supernatant was removed, and new culture fluid was added for continued culture. The second to fifth-generation cells were obtained for the following experiments.

### Apoptosis detection

Apoptotic cells were detected using Terminal deoxynucleotidyl transferase dUTP nick end labeling (TUNEL; KGI Biotechnology Development Co., Ltd., Nanjing, China), according to the manufacturer’s instructions, and were observed using an IX81 fluorescence microscope (Olympus Corporation, Tokyo, Japan).

Apoptosis was also detected by flow cytometry (FACSCanto; BD Biosciences, Inc., Franklin Lakes, NJ, USA). The cells (5×10^3^/ml) were collected by trypsin digestion, centrifuged to precipitate the cells at 200 x g for 5 min at 4°C and washed twice with PBS, prior to fixing in 2 ml 70% ethanol (Sigma-Aldrich) at 4°C overnight. The cells were washed twice with PBS, as described above, and 200 ml RNaseA (Fuzhou Maixin Biotechnology Development Co., Ltd.) at a final concentration of 25 g/ml, was added to the cells and incubated at 37°C in a water bath (DFY-10; Changzhou JieBo sen Instrument Co., Ltd., Changzhou, China) for 30 min. Propidium iodide (PI; Sigma-Aldrich) solution (500 ml) at a concentration of 50 g/ml was added to the cells for 30 min at 4°C in the dark, prior to being filtered using a 300 mesh nylon mesh (Shanghai Tesheng Filters Material Co., Ltd., Shanghai, China) and detected by flow cytometry. The number of detected cells required was ≥5×10^4^, the excitation wavelength was 488 nm and the emission wavelength was 620 nm. A histogram plotting the intensity of the PI signal and a scattergram demonstrating the front scattered light against the side-scattered light were plotted using CellQuest software (BD Biosciences, Inc.).

### Total cellular protein extracts

Following treatment, the cell culture was removed and the cells (5×10^3^/ml) were gently washed three times with PBS. The adherent cells were scraped off the culture plates using a cell scraper and collected by centrifugation at 1,049 x g for 3 min at 4°C. The supernatant was carefully removed and 100 ml lysis buffer (1 volume of sediment:3 volumes of buffer), pre-cooled at 0°C, was added at a volume three times that of the volume of sedimentation. The cells were repeatedly pipetted (5 times) and the cell suspension was disrupted by agitation with glass beads (0.4–0.5 mm) at 4°C for 30 min. The cell lysates were collected and centrifuged at 1,049 x g for 10 min at 4°C and the supernatant, containing the total cellular protein was preserved at −80°C for subsequent use.

### Caspase-3 activity assay

The cells (5×10^3^/ml) from each experimental group (0, 0.01, 0.1, 1, 10, 100 and 1,000 *µ*mol/l aldosterone treatment) were collected, washed twice with PBS and the total cell protein was obtained by dissociating and extracting, as mentioned in the previous paragraph. Reaction buffer (2X), containing 10 mM dithiothreitol and 1 mM caspase-3 tetrapeptide fluorogenic substrate (acetyl Asp-Glu-Val-Asp 7-amido-4-methylcoumarin; Ac-DEVD-AMC) (Fuzhou Maixin Biotechnology Development Co., Ltd.) was added to the protein lysate and incubated in 96-well plates at 37°C to react for 60 min. Fluorescence analysis was performed using a DG5033A microplate reader (Shanghai Precision & Scientific Instrument Co., Ltd., Shanghai, China) with an excitation wavelength of 380 nm and an emission wavelength of 430–460 nm. Depending on the fluorescence intensity of AMC, the activity of caspase-3 was measured to reflect the degree of activated caspase-3.

### PARP activity detection

The activity of PARP was detected in cultured endothelial cells using a PARP Detection kit (Trevigen, Gaithersburg, MD, USA), according to the manufacturer’s instructions. The assessment required at least three repeats for each sample to ensure reliable detection results.

### RNA extraction and reverse transcription

The first-strand cDNA was generated using a TransScript First-Strand cDNA Synthesis SuperMix kit (Transgen, Beijing, China). The first-strand cDNA was diluted 10-fold as a template for polymerase chain reaction (PCR) or quantitative (q)PCR.

### qPCR

The qPCR was performed following the addition of SYBR premix EX taq kit (Takara Bio Inc., Otsu, Shiga, Japan) and gently mixing the plates. The quantity of the cDNA sample was 2 *µ*l, the primer sequences used are presented in Table I and the cycling conditions were as follows: 40 cycles of 95°C for 30 sec, 95°C for 5 sec and 60°C for 34 sec.

### Immunoblotting

The proteins were separated by molecular weight using 10% SDS-PAGE gels (Sigma-Aldrich) and were transferred onto a nitrocellulose membrane (Advantec MFS, Inc., Dublin, CA, USA). The membrane was subsequently blocked in 5% non-fat milk at room temperature for 60 min.

The membranes were then incubated with the primary antibody (rabbit anti-human PARP polyclonal antibody; #9664; Cell Signaling Technology, Inc., MA, USA) diluted in 5% non-fat milk (Fuzhou Maixin Biotechnology Development Co., Ltd.) overnight at 4°C. Following washing in Tris-buffered saline containing Tween-20 (TBST; Shanghai Sangon, Biological Engineering Co., Ltd.) three times for 10 min on a horizontal shaker (HZ-9611K; Hualida Experiment Equipment Company, Nanjing, China), the membranes were incubated with the secondary antibody (horseradish-peroxidase-labeled goat anti-rabbit IgG; Beijing Zhongshan Golden Bridge Biotechnology Co., Ltd., Beijing, China) diluted in 5% non-fat milk at room temperature for 60 min and were washed with TBST and stained with DAB coloration liquid (Sigma-Aldrich), followed by developing with the ECL system (GE Healthcare Life Sciences, Livingston, NJ, USA) according to the manufacturer’s instructions.

## Results

### Effect of different concentrations of aldosterone on apoptosis

The subcultured HUVECs were treated with aldosterone (0, 0.01, 0.1, 1, 10, 100 and 1,000 *µ*mol/l; Fluka, St. Louis, MO, USA) for 48 h. The 0 *µ*mol/l concentration was used as the control group. Following treatment, the apoptotic cells were detected by a variety of methods.

**Figure 1:**
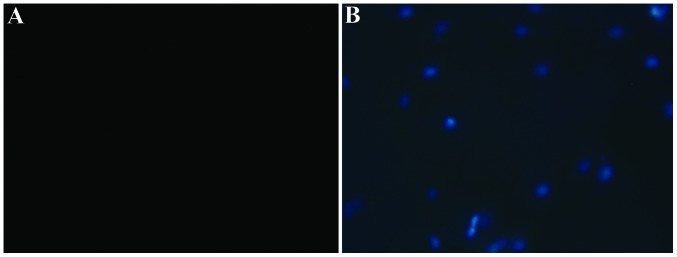
Detection of apoptosis by terminal deoxynucleotidyl transferase dUTP nick end labeling in the (A) control group (0 *µ*mol/l aldosterone) and (B) aldosterone (1,000 *µ*mol/l aldosterone) group. Staining was observed using a fluorescence microscope; magnification, x400. No staining was observed in (A), whilst (B) demonstrated an increased number of apoptotic cells.

TUNEL was used to determine the number of endothelial cells undergoing apoptosis. No positive staining was detected following treatment of the HUVECs with 0, 0.01, 0.1, 1 or 10 *µ*mol/l aldosterone, similar to the control group. However, when the concentration of aldosterone increased to 100 *µ*mol/l, a number of cells exhibited positive TUNEL staining, indicating that free 3′ hydroxyl groups were produced due to DNA cleavage in these cells, suggesting that several cells have entered apoptosis. When the concentration was further increased to 1,000 *µ*mol/l, the number of positive TUNEL stained cells increased, indicating that a number of cells had entered the apoptotic process (Fig. 1).

**Figure 2:**
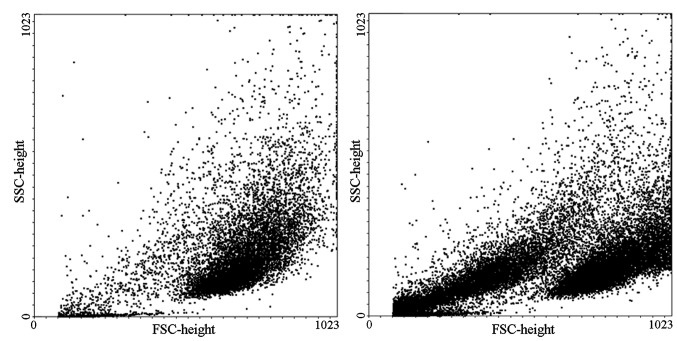
Analysis of apoptosis by measuring light scattering changes by flow cytometry. Left (0 *µ*mol/l aldosterone), no clear cell apoptosis was observed; right (1,000 *µ*mol/l aldosterone), clear cell apoptosis observed. SSC, side scatter; FSC, forward scatter.

Flow cytometry was used to detect and quantify the number of apoptotic cells. The results demonstrated that dense endothelial cells treated with low concentrations of aldosterone (0.01, 0.1, 1 and 10 *µ*mol/l) exhibited no differences in the number of apoptotic cells compared with the 0 *µ*mol/l control group. When the concentration was increased to 100 *µ*mol/l, a marked increase of apoptosis was observed compared with the control group (0.64, vs. 18.3%; P<0.01). The effect on apoptosis was more pronounced when the concentration of aldosterone was increased to 1,000 *µ*mol/l compared with the control group (0.64, vs. 42.5%; P<0.01; Fig. 2).

### Effect of aldosterone treatment duration on apoptosis

The subcultured HUVECs were treated with aldosterone at a final concentration of 1,000 *µ*mol/l for 24, 48 or 72 h. An untreated group of cells was used as a control.

**Figure 3:**
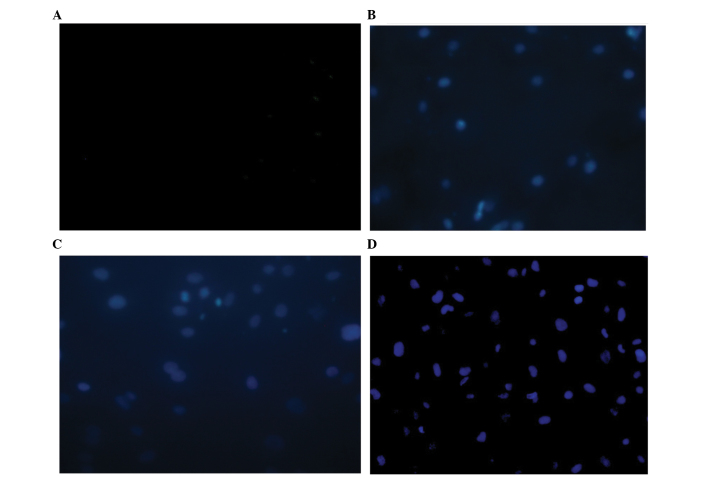
Detection of apoptosis by terminal deoxynucleotidyl transferase dUTP nick end labeling in the (A) control group (0 *µ*mol/l aldosterone), (B) 24 h group (1,000 *µ*mol/l aldosterone), (C) 48 h group (1,000 *µ*mol/l aldosterone) and (D) 72 h group (1,000 *µ*mol/l aldosterone) (magnification, x400). No staining was observed in control group. The number of positive TUNEL stained cells increased with the treatment time.

TUNEL was used to determine endothelial cell apoptosis. When the duration of aldosterone treatment reached 24 h, a small quantity of TUNEL-positive cells were detected. The number of positive TUNEL stained cells further increased at 48 h and markedly increased until 72 h (Fig. 3).

Apoptosis was also detected and quantified using flow cytometry. The results demonstrated that the ability of aldosterone (1,000 *µ*mol/l) to induce the apoptosis of endothelial cells with high cell density increased with increasing treatment durations. When the treatment duration reached 72 h, the majority of cells had died and the number of cells was significantly less compared with any of the other experimental groups, suggesting that several cells had been lysed into cell fragments, which cannot be used for further PI staining or fluorescent activated cell sorting analysis.

### Effects of aldosterone on the activity of caspase-3

The effect of aldosterone at different concentrations on the activity of caspase-3 in cells was investigated. On the basis of the preceding detection result of cell apoptosis, the cells were treated with aldosterone (100 or 1,000 *µ*mol/l) for 48 h at different cell densities and the cells (5×10^3^/ml) were collected and dissociated prior to extracting the total cellular protein for a caspase-3 activity assay.

The caspase-3 activity results from the treatment of HUVECs at higher or lower densities were consistent with the results from the apoptotic assays. The cells treated with 100 *µ*mol/l aldosterone increased the activity of caspase-3 and, following treatment with 1,000 *µ*mol/l aldosterone, a more marked increase in the activity of caspase-3 was observed. The activity of caspase-3 was more markedly increased in the high cell density group compared with the low cell density group.

The effect of aldosterone treatment duration on the vitality of caspase-3 in HUVECs was also investigated. According to the apoptosis results, the cells were detected at lower densities and treated with 1,000 *µ*mol/l aldosterone for 24, 48 or 72 h. The cells were collected and dissociated at the end of the treatment and the total cellular protein was extracted for the detection of caspase-3 activity. The results were consistent with those of apoptosis. Caspase-3 activity increased as the treatment duration increased and the rate of increased gradually.

### Effects of aldosterone treatment on the activity of PARP1

The effect of different concentrations of aldosterone on the activity of intracellular PARP1 was subsequently investigated. Based on the preceding results of apoptosis, the cells were treated with 100 and 1,000 *µ*mol/l aldosterone for 48 h at different cell densities, and the cells were collected, dissociated and the total cellular protein was extracted to detect PARP1 activity. Treatment with 100 *µ*mol/l aldosterone upregulated the activity of PARP1, while 1,000 *µ*mol/l aldosterone treatment upregulated the activity of PARP1 more markedly. The increased range of PARP1 activity were increased in the cells at a high density compared with those at a low density.

**Figure 4:**
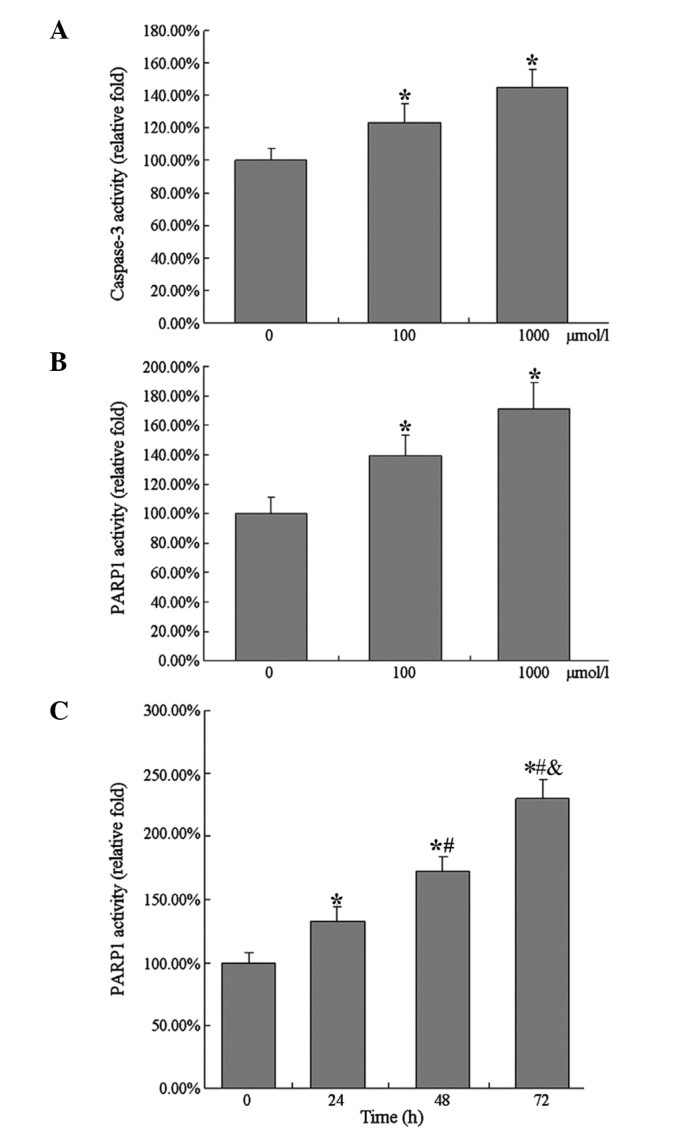
Caspase-3 and PARP1 activity following treatment with aldosterone. (A) Caspase-3 activity assay (high cell density) and aldosterone. (B) PARP1 activity assay (high cell density) following treatment with aldosterone. (C) Detection of PARP1 activity following treatment with aldosterone for different durations. The error bars represented standard deviation. ^*^P<0.05 vs. control group, ^#^P<0.05 vs. 24 h group and ^&^P<0.05 vs. 48 h group. PARP, poly (ADP-ribose) polymerase.

The effect of the duration of aldosterone treatment on the activity of PARP1 in HUVECs was also assessed. According to the results of apoptosis, the cells were selected to be detected at lower densities, treated with 1,000 *µ*mol/l aldosterone for 24, 48 or 72 h. The cells were collected and dissociated following treatment, and the total cellular protein was extracted for detection of PARP1 activity. As the duration of treatment increased, the vitality of PARP1 increased and this rate of increase was gradual (Fig. 4).

### Inhibition of eplerenone on the activation of caspase-3 by aldosterone

Processing HUVECs at a lower cell density and treating with 1,000 *µ*mol/l eplerenone completely inhibited the activation of caspase-3 by aldosterone. However, in the case of high cell density, 1,000 *µ*mol/l eplerenone did not completely suppress the activation of caspase-3 by aldosterone and only partially reduced the activation of caspase-3.

### Inhibition of eplerenone on the activation of PARP1 by aldosterone

Processing HUVECs at a lower cell density and treating with 1,000 *µ*mol/l eplerenone completely inhibited the activation of PARP1 by aldosterone. However, in the case of high cell density, 1,000 *µ*mol/l eplerenone did not completely suppress the activation of PARP1 by aldosterone and only partially reduced the activation of PARP1.

### Detection of the protein expression of PARP1

Endothelial cells cultured at a low growth density were treated with 1,000 *µ*mol/l eplerenone and/or 1,000 *µ*mol/l aldosterone for 48 h and cells without any treatment were used as a control group. The cells were collected following treatment and were divided into two groups, one for extracting RNA to determine the mRNA expression of PARP1 by qPCR, and another for extracting total cellular protein to determine the expression of PARP1 by immunobloting.

**Figure 5:**
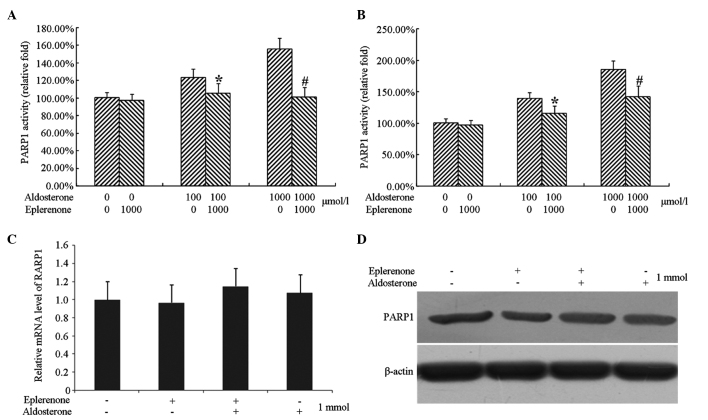
PARP1 activity following treatment with eplerenone and aldosterone. (A) Detection of PARP1 activity (low cell density), (B) detection of PARP1 activity (high cell density), (C) mRNA expression of PARP1 and (D) protein expression of PARP1 following treatment with aldosterone and/or eplerenone.^*^P<0.05 vs. 100 *µ*mol/l aldosterone group, ^#^P<0.05 vs. 1,000 *µ*mol/l aldosterone group. PARP, poly (ADP-ribose) polymerase.

The results revealed that neither treatment of cells with eplerenone or aldosterone alone or together affected the mRNA and protein expression levels of PARP1 (Fig. 5).

### Inhibition of ABT-888 on aldosterone-induced apoptosis

The subcultured HUVECs were treated with aldosterone at a final concentration of 0, 100 or 1,000 *µ*mol/l in the corresponding cell wells as a control group and ABT-888 (Sigma-Aldrich) was added to the experimental group. Following culturing for 48 h, the culture supernatant from the cells was collected and all the adherent cells were digested using trypsin, aggregated and fixed with 70% ethanol, prior to staining with PI for flow cytometric analysis.

**Figure 6:**
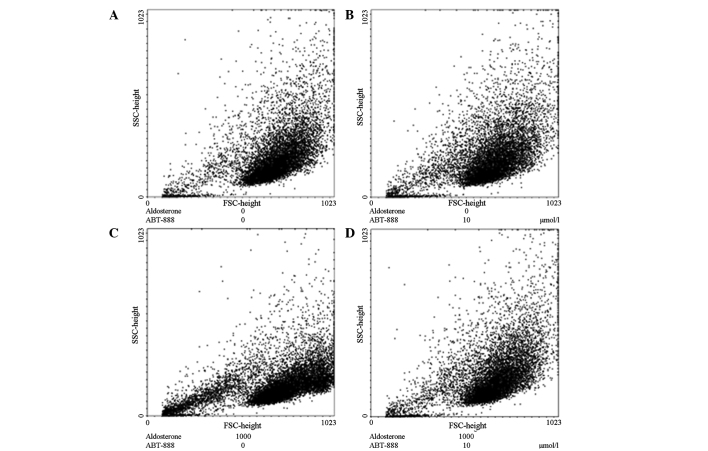
Detection of apoptosis by flow cytometry following treatement with various concentrations of aldosterone and ABT-888. (A) Untreated, (B) treatment with ABT-888 (10 *µ*mol/l), (C) treatment with aldosterone (1,000 *µ*mol/l) and (D) treatment with ABT-888 (10 *µ*mol/l) and aldosterone (1,000 *µ*mol/l). FSC, forward scatter; SSC, side scatter.

The results demonstrated that the cells treated with aldosterone at a final concentrations of 100 and 1,000 *µ*mol/l induced apoptosis significantly, which was consistent with the previous results. Treatment with ABT-888 (10 *µ*mol/l) and aldosterone (100 and 1,000 *µ*mol/l), completely inhibited aldosterone-induced apoptosis (Fig. 6).

### ABT-888 inhibits the activation of caspase-3 by aldosterone

Based on the results demonstrating that ABT-888 inhibited aldosterone-induced apoptosis, low density cells were treated with aldosterone (100 or 1,000 *µ*mol/l) and ABT-888 (10 *µ*mol/l) for 48 h. The cells were collected and dissociated following treatment, and the total cellular protein was extracted for caspase-3 activity assay.

The results of caspase-3 activity were consistent with the apoptosis, revealing that treatment with 100 *µ*mol/l aldosterone increased that activity of caspase-3. A more marked increase in the activity of caspase-3 was observed following treatment with 1,000 *µ*mol/l aldosterone. Treatment with 10 *µ*mol/l ABT-888, led to complete inhibition of the activation of caspase-3 by aldesterone.

### Impact of ABT-888 on PARP1 activity

To confirm the inhibitory effect on the cell viability PARP1 following treatment with ABT-888, the treated cells were collected, dissociated and the total cell protein was extracted to detect PARP1 activity.

**Figure 7:**
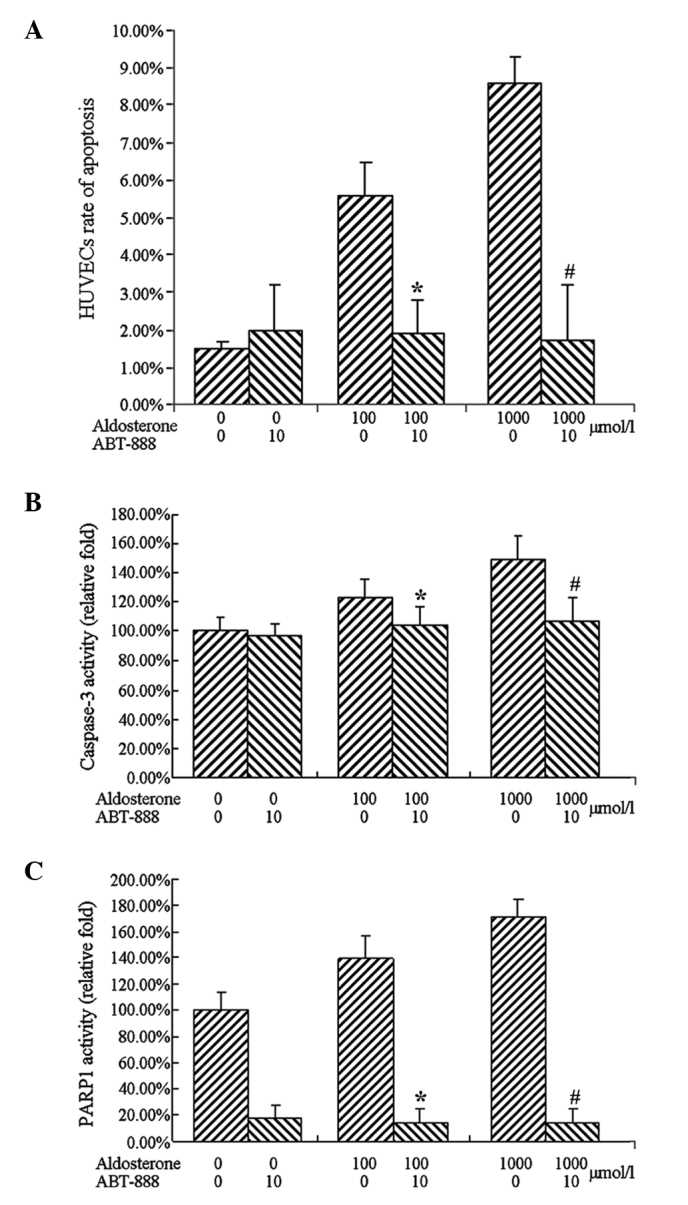
Analysis of (A) apoptotic cells detected by flow cytometry via scattered light changes following treatment with ABT-888-aldosterone. Analysis of (B) caspase-3 activity and (C) PARP1 activity following treatment with ABT-888-aldosterone. ^*^P<0.05 vs. 100 *µ*mol/l aldosterone group, ^#^P<0.05 vs. 1,000 *µ*mol/l aldosterone group. HUVECs, human umbilical vein endothelial cells; PARP, poly (ADP-ribose) polymerase.

Treatment with 100 or 1,000 *µ*mol/l aldosterone upregulated the activity of PARP1 and cotreatment with 10 *µ*mol/l ABT-888 significantly reduced PARP1 activity (Fig. 7).

## Discussion

The activity of the PARP1 protease is catalyzed by poly ADP-ribosyl in eukaryotic cells and this protein is involved in DNA repair following DNA damage or fracture. The PARP enzyme activity accounts for >90% of the enzyme activity in the protein family (19). PARP1 is important in DNA repair and apoptosis. Deletion of PARP1 renders cells more sensitive to DNA damage and causes a marked accumulation of genetic mutations, inducing tumorigenesis (20,22).

The accumulation of DNA damage often leads to cancer and genetic mutations, whereas excessive DNA damage can lead to apoptosis. Breast cancer susceptibility gene (BRCA)1 and BRCA2 are DNA double-strand break repair proteins and deletion of either protein causes the accumulation of DNA mutations, leading to breast cancer. PARP1 inhibitors are used to treat breast cancer exhibiting deletion of BRCA1 or BRCA2. With this deletion, a large number of cells exhibiting DNA damage and inhibiting the activity of PARP1 further increased DNA damage, leading to apoptosis and ultimately treating tumors (23).

Numerous PARP1-specific inhibitors are available for the clinical treatment of cancer. Previous studies have demonstrated that elevated PARP1 activity in normal cells leads to damage of endothelial function (24). The present study confirmed that the abnormal elevation of plasma aldosterone concentration induced apoptosis of endothelial cells, thereby triggering endothelial dysfunction. It was demonstrated that this effect was transmitted into the cells by aldosterone receptors. Aldosterone also increased the activity of PARP1 in cells, however, the activation of PARP1 was not caused by regulating mRNA or protein expression levels.

The PARP1 inhibitor, ABT-888, is commonly used with aldosterone to process cells and it has been revealed that the increase in PARP1 activity mediated the effect of aldosterone on the apoptosis of endothelial cells. Specific concentrations of ABT-888 reduced PARP1 activity to low levels and completely inhibited the aldosterone-stimulated apoptosis of HUVECs.

ABT-888 also acted on the nucleoprotein PARP1 directly through the cell membrane, this confirmed the effect of aldosterone stimulating apoptosis through the aldosterone receptor. The stimulation of aldosterone was transferred between the extracellular and intracellular environment through its receptors, while ABT-888 directly inhibited intracellular PARP1 activity. Inhibition of intracellular PARP1 protein inhibited the activity of the extracellular signal, which confirmed that PARP1 apoptosis was mediated by aldosterone.

The present study investigated the molecular mechanisms underlying aldosterone-induced endothelial cell damage through the activation of PARP1 activity, leading to cell apoptosis. However, the mechanisms underlying the activation of PARP1 by the aldosterone activating aldosterone receptor requires further investigation. It was found that the activation and role of aldosterone on PARP1 was not due to altering the mRNA or protein levels and, therefore, the mechanisms require further investigation.

The present study confirmed that the PARP1 inhibitor, ABT-888, inhibited the effect of high concentrations of aldosterone on the injury of cultured endothelial cells, suggesting a novel direction in the treatment of cardiovascular diseases caused by aldosterone. However, *in vitro* experiments do not completely simulate *in vivo* conditions, and the feasibility of the treatment programs also require further investigation in animal experiments.

## Figures and Tables

**Table I:** Primer sequences used for reverse transcription quantitative polymerase chain reaction.

Target gene	Forward primer (5′-3′)	Reverse primer (5′-3′)
β-actin	GTTGTCGACGACGAGCG	GCACAGAGCCTCGCCTT
Poly (ADP-ribose) polymerase 1	TCTGCCTTGCTACCAATTCC	GATGGGTTCTCTGAGCTTCG
